# NSF is required for diverse endocytic modes by promoting fusion and fission pore closure in secretory cells

**DOI:** 10.1016/j.isci.2026.115510

**Published:** 2026-03-27

**Authors:** Xin-Sheng Wu, Tao Sun, Bo Shi, Sunghoon Lee, Zheng Zhang, Lisi Wei, Xin Wang, Maryam Molakarimi, Sue Han, Aaron Uy, Lin Gan, Ling-Gang Wu

**Affiliations:** 1 National Institute of Neurological Disorders and Stroke, 35 Convent Dr, Bldg. 35, Rm. 2B-1012, Bethesda, MD 20892, USA; 2 Flaum Eye Institute, University of Rochester School of Medicine and Dentistry, Rochester, New York, NY 14642, USA

**Keywords:** Neuroscience, Cellular neuroscience

## Abstract

The ATPase N-ethylmaleimide-sensitive factor (NSF), known for disassembling SNARE complexes, plays key roles in neurotransmitter release, neurotransmitter (AMPA, GABA, and dopamine) receptor trafficking, and synaptic plasticity, and its dysfunction or mutation is linked to neurological disorders. These roles are largely attributed to SNARE-mediated exocytosis. Here, we reveal an unexpected role for NSF: mediating diverse modes of endocytosis—including slow, fast, ultrafast, overshoot, and bulk—by driving closure of both fusion and fission pores. This function was consistently observed across large calyx nerve terminals, small hippocampal boutons, and chromaffin cells using capacitance recordings, synapto-pHluorin imaging, electron microscopy, and multi-color pore-closure imaging. Results were robust across four NSF inhibitors, gene knockout, knockdown, and mutations. Furthermore, NSF facilitates content release. These findings establish NSF as a central regulator of membrane fission, kiss-and-run fusion, endocytosis, and exo-endocytosis coupling, providing a mechanistic basis for its diverse roles in synaptic transmission, receptor trafficking, and disease.

## Highlights

• NSF is essential for slow, fast, ultrafast, overshoot, and bulk endocytosis
• NSF promotes endocytosis by closing fusion pores and pre-existing Ω-profiles’ pore
• NSF promotes pore closures (including kiss-and-run) by disassembling SNARE complex
• NSF facilitates vesicular content release

## Graphical abstract

**Figure:**
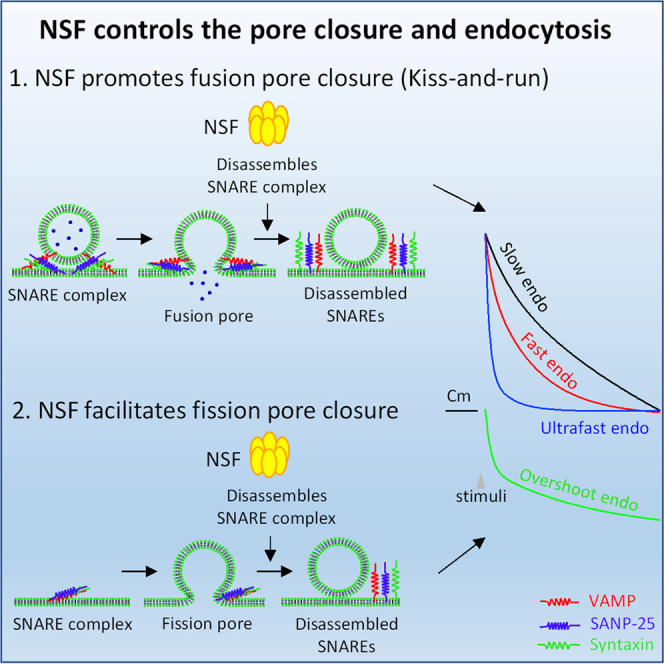

## Introduction

The ATPase N-ethylmaleimide-sensitive factor (NSF) is well-known to disassemble the soluble NSF attachment protein receptor (SNARE) complex, composed of synaptobrevin, SNAP-25, and syntaxin, which drives vesicle fusion, releasing neurotransmitters and hormones. Genetic loss of NSF results in the accumulation of *cis*-SNARE complexes on synaptic vesicles and inhibition of transmitter release in Drosophila synapses.^1^ Acute peptide-based perturbation experiments in giant squid synapses show that impaired NSF activity causes a rapid block in neurotransmission and slowing down of release, suggesting that SNARE complex disassembly is crucial for transmitter release at the pre-fusion state.^2^^,^^3^ Mutation and pharmacological inhibition of NSF reveal NSF in regulating vesicle priming for fusion at release sites in Drosophila synapses and mouse hippocampal synapses,^4^^,^^5^ suggesting that the SNARE complex disassembly is important for vesicle priming at the pre-fusion state. Besides regulating vesicle fusion at the nerve terminal, NSF has been shown to regulate the trafficking of important neurotransmitter receptors, such as AMPA receptors, GABA receptors, and dopamine receptors, thereby regulating synaptic plasticity.^6^ Aggregation, functional impairment, and/or mutation of NSF have been linked to major neurological disorders, including Parkinson’s disease, Alzheimer’s disease, and epilepsy.^6^ In brief, NSF is crucial for transmitter release, neurotransmitter receptor trafficking, synaptic plasticity, and neurological disorders. These diverse physiological functions and pathological roles have been largely attributed to its ability to disassemble the SNARE complex, crucial for vesicle priming and release.^6^

Little is known about whether NSF plays any role after vesicle fusion, such as the fusion pore expansion and closure, and subsequent endocytosis that recycles exocytosed vesicles to sustain synaptic transmission and exocytosis in secretory cells. Here, we studied whether NSF is involved in these post-fusion roles by (1) measuring slow, fast, and ultrafast endocytosis with capacitance measurements in chromaffin cells and calyx of Held synapses, (2) detecting endocytosis with synapto-pHluorin imaging in hippocampal synapses, and (3) quantifying bulk endocytosis with electron microscopy (EM) in hippocampal synapses. By inhibiting NSF with various pharmacological inhibitors, gene knockout or knockdown, we found, to our surprise, that NSF is essential for each of these different forms of endocytosis in three preparations. By imaging fusion pore opening and closure, as well as the fission pore closure, we found that NSF is crucial in mediating diverse forms of endocytosis mentioned above by playing a critical role in closing both fusion and fission pores in chromaffin cells. These results suggest including NSF as a key player in the current models of membrane fission, diverse modes of endocytosis, vesicle recycling, and exo-endocytosis coupling, which may contribute to accounting for NSF’s diverse physiological and pathological roles discussed above.

## Results

### NSF is involved in slow and fast endocytosis at calyces

#### Recording conditions

**Figure 1:**
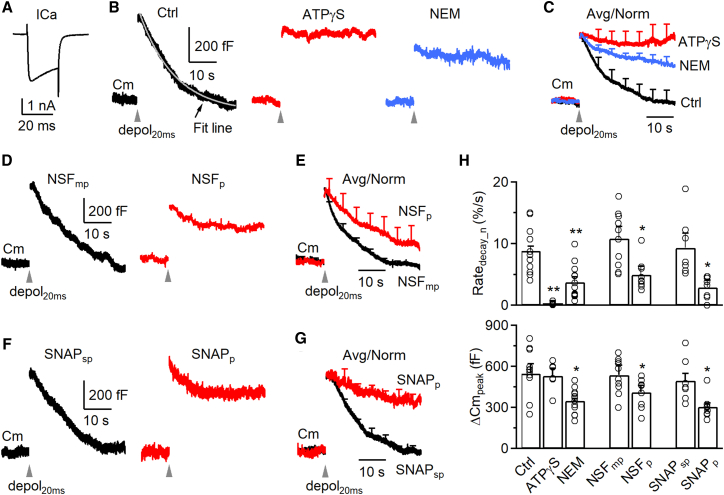
NSF is involved in slow endocytosis at calyces (A) Sampled calcium current (ICa) induced by depol_20ms_ in a calyx of Held. Vertical scale bar, 1 nA; horizontal scale bar, 20 ms. (B) Sampled single traces showing membrane capacitance (Cm) changes induced by depol_20ms_ (gray arrowheads) at 4–10 min after break-in with a pipette containing a control solution (Ctrl, black), 4 mM ATPγS (replacing ATP) (red), or 1 mM NEM (blue). The Cm decay in Ctrl was fitted with a mono-exponential function (left, fit line in gray, τ = 10.6 s). Vertical scale bar, 200 fF; horizontal scale bar, 10 s. Scale bars apply to all traces in (B). (C) Averaged traces showing capacitance changes induced by depol_20ms_ (gray arrowhead) at 4–10 min after break-in with a pipette containing a control solution (black, 11 calyces, from 5 male and 6 female rats), 4 mM ATPγS (replacing ATP, red, 6 calyces, from 3 male and 3 female rats) or 1 mM NEM (blue, 12 calyces, from 6 male and 6 female rats). The peak amplitude of the ΔCm_peak_ was normalized (Avg/Norm), and data are expressed as mean + s.e.m. every 5 s (applies also to E and G). Horizontal scale bar, 10 s. (D and E) Similar arrangements as in (B) and (C), respectively, but with NSF_mp_ (1 mM, black, Ctrl, 10 calyces, from 5 male and 5 female rats) or NSF_p_ (1 mM, red, 9 calyces, from 4 male and 5 female rats). Vertical scale bar, 200 fF; horizontal scale bars, 10 s. (F–G) Similar arrangements as in (D) and (E), respectively, but with SNAP_sp_ (1 mM, black, Ctrl, 7 calyces, from 3 male and 4 female rats) or SNAP_p_ (1 mM, red, 8 calyces, from 4 male and 4 female rats). Vertical scale bar, 200 fF; horizontal scale bars, 10 s. (H) The Rate_decay_n_ (upper) and ΔCm_peak_ (lower) induced by depol_20ms_ at 4–10 min after break-in with a pipette containing the control solution (Ctrl, 11 calyces, from 5 male and 6 female rats), ATPγS (4 mM, 6 calyces, from 3 male and 3 female rats), NEM (1 mM, 12 calyces, from 6 male and 6 female rats), NSF_mp_ (1 mM, 10 calyces, from 5 male and 5 female rats), NSF_p_ (1 mM, 9 calyces, from 4 male and 5 female rats), SNAP_sp_ (1 mM, 7 calyces, from 3 male and 4 female rats), or SNAP_p_ (1 mM, 8 calyces, from 4 male and 4 female rats). Data are expressed as mean + s.e.m. Each circle represents the data from a single calyx. ∗*p* < 0.05; ∗∗*p* < 0.01 (*t* test). See also Figures S1 and S2.

**Figure 2:**
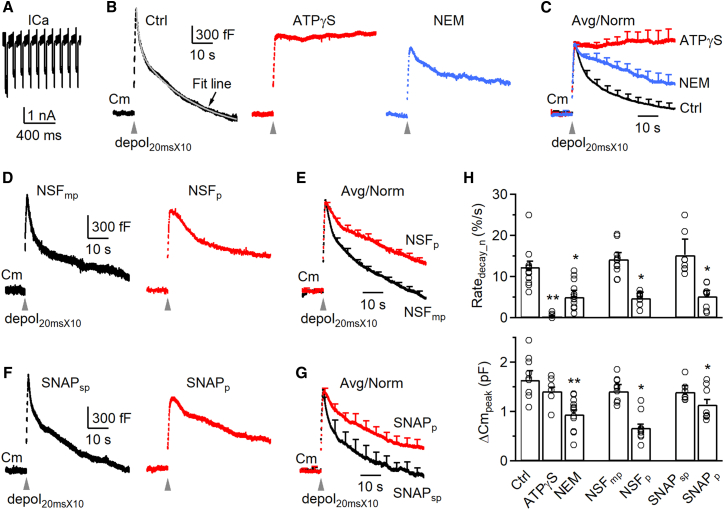
NSF is involved in fast endocytosis at calyces (A) Sampled calcium current (ICa) induced by depol_20msX10_ in a calyx of Held. Vertical scale bar, 1 nA; horizontal scale bar, 400 ms. (B) Sampled single traces showing membrane capacitance (Cm) changes induced by depol_20msX10_ (gray arrowheads) at 4–10 min after break-in with a pipette containing a control solution (black, Ctrl), 4 mM ATPγS (replacing ATP) (red) or 1 mM NEM (blue). The Cm decay in Ctrl was fitted with a bi-exponential function (left; gray fit line) with τ_1_ = 1.4 s (weight: 29%) and τ_2_ = 18.3 s. Vertical scale bar, 300 fF; horizontal scale bar, 10 s. Scale bars apply to all traces in (B). (C) Averaged traces showing capacitance changes induced by depol_20msX10_ (gray arrowhead) at 4–10 min after break-in with a pipette containing a control solution (black, 11 calyces, from 5 male and 6 female rats), 4 mM ATPγS (replacing ATP, red, 6 calyces, from 3 male and 3 female rats), or 1 mM NEM (blue, 12 calyces, from 6 male and 6 female rats). The peak amplitude of the ΔCm_peak_ is normalized (Avg/Norm), and data are expressed as mean + s.e.m. every 5 s (applies also to E and G). Horizontal scale bar, 10 s. (D and E) Similar arrangements as in (B) and (C), respectively, but with NSF_mp_ (1 mM, black, Ctrl, 10 calyces, from 5 male and 5 female rats) or NSF_p_ (1 mM, red, 9 calyces, from 4 male and 5 female rats). Vertical scale bar, 300 fF; horizontal scale bars, 10 s. (F and G) Similar arrangements as in (D) and (E), respectively, but with SNAP_sp_ (1 mM, black, Ctrl, 7 calyces, from 3 male and 4 female rats) or SNAP_p_ (1 mM, red, 8 calyces, from 4 male and 4 female rats). Vertical scale bar, 300 fF; horizontal scale bars, 10 s. (H) The Rate_decay_n_ (upper) and ΔCm_peak_ (lower) induced by depol_20msX10_ at 4–10 min after break-in with a pipette containing the control solution (Ctrl, 11 calyces, from 5 male and 6 female rats), ATPγS (4 mM, 6 calyces, from 3 male and 3 female rats), NEM (1 mM, 12 calyces, from 6 male and 6 female rats), NSF_mp_ (1 mM, 10 calyces, from 5 male and 5 female rats), NSF_p_ (1 mM, 9 calyces, from 4 male and 5 female rats), SNAP_sp_ (1 mM, 7 calyces, from 3 male and 4 female rats), or SNAP_p_ (1 mM, 8 calyces, from 4 male and 4 female rats). Data are expressed as mean + s.e.m. Each circle represents data from a single calyx. ∗*p* < 0.05; ∗∗*p* < 0.01 (*t* test). See also Figure S2.

We measured slow (Figure 1) and fast (Figure 2) endocytosis by whole-cell capacitance recordings at calyces with a pipette containing either a control solution or one of the following four types of NSF (ATPase) inhibitors: (1) ATPγS (replacing ATP, 4 mM, *n* = 6) or 0 ATP (*n* = 7), (2) N-ethylmaleimide (NEM, 1 mM, *n* = 12), (3) an NSF peptide (NSF_p_, 1 mM, *n* = 9),^3^ and (4) an SNAP peptide (SNAP_p_, 1 mM, *n* = 8) that blocks the binding between NSF and SNAP (soluble NSF attachment protein), a chaperone for recruiting NSF.^7^ The corresponding control solution for peptides contained either mutated NSF_p_ (NSF_mp_, 1 mM, *n* = 10) or scrambled SNAP_p_ (SNAP_sp_, 1 mM, *n* = 7).

#### Quantifying slow and fast endocytosis in control

We first analyzed data during 4–10 min after whole-cell break-in. In control, we induced slow and fast endocytosis with 1 and 10 pulses of 20 ms depolarization (from −80 to +10 mV, if not mentioned otherwise) at 10 Hz, called depol_20ms_ (Figure 1A) and depol_20msX10_ (Figure 2A), respectively.^8^^,^^9^^,^^10^ A depol_20ms_ induced a capacitance jump (ΔCm_peak_) of 531 ± 55 fF (*n* = 11), followed by a slow decay with a τ of 10.2 ± 0.9 s (*n* = 11) and an initial decay rate (Rate_decay_), measured during 0.5–4 s after depol_20ms_ (see also “STAR Methods”), of 56 ± 9 fF/s (*n* = 11, e.g., Figure 1B, left). A depol_20msX10_ induced a ΔCm_peak_ of 1,565 ± 115 fF (*n* = 11), followed by a bi-exponential decay with a rapid τ of 1.6 ± 0.3 s (amplitude: 31 ± 4%) and a slow τ of 15.5 ± 1.4 s (*n* = 11, e.g., Figure 2B). The Rate_decay_, measured during 0.5–1.5 s after depol_20msX10_ (see also “STAR Methods”), was 301 ± 55 fF/s (*n* = 11, e.g., Figure 2B, left), which reflected mostly (>80%) the rapid component of endocytosis as demonstrated previously.^8^^,^^9^^,^^10^ When NSF_mp_ or SNAP_sp_ was included, serving as the control for NSF_p_ or SNAP_p_, it did not significantly affect the results described above (Figures 1D–1H and 2D–2H). These control results were similar to previous reports.^8^^,^^9^^,^^10^

Rate_decay_ at calyces was measured between 0.5 and 4 s after depol_20ms_ that induced slow endocytosis, but between 0.5 and 1.5 s after depol_20msX10_ that induced rapid endocytosis.^9^^,^^10^^,^^11^ Rate_decay_n_ was measured as Rate_decay_ divided by the capacitance jump peak amplitude (ΔCm_peak_).

When ΔCm_peak_ was normalized to 1, the initial rate of Cm decay (Rate_decay_n_, see “STAR Methods” for calculation) after depol_20ms_ was 0.09 ± 0.01/s (*n* = 11), meaning 9% of ΔCm_peak_ was retrieved at the first second after stimulation. The Rate_decay_n_ after depol_20msX10_ (see “STAR Methods” for calculation) was 0.12 ± 0.01/s (*n* = 11), which largely reflected the normalized rapid endocytosis rate.^8^^,^^9^^,^^10^ Throughout the study, we compared these normalized values (Rate_decay_n_) in control and in the presence of drugs. We did not compare τ, because τ was often too long to estimate in the presence of blockers (Figures 1 and 2). We did not compare Rate_decay_, because it is influenced by ΔCm_peak_, which was often reduced by the tested blockers.

#### Inhibition of NSF inhibits both slow and fast endocytosis

At 4–10 min after whole-cell break-in, four NSF blockers dialyzed via the whole-cell pipette into the calyx, including ATPγS, NEM, NSF_p_, and SNAP_p_, substantially reduced the Rate_decay_n_ measured after depol_20ms_ (Figures 1B–1H) or depol_20msX10_ (Figures 2B–2H) by more than 60%, but did not affect the calcium current amplitude (ICa; Figure S1). Consequently, these four blockers substantially prolonged the normalized, averaged capacitance decay (Figures 1B–1G and 2B–2G). These results suggest that NSF is involved in mediating both slow and fast endocytosis (Figures 1 and 2).

#### Rate_decay_n_ reduction is independent of ΔCm_peak_ reduction

Except for ATPγS and 0 ATP, other blockers reduced ΔCm_peak_ to a value >60% of control (Figures 1B–1H and 2B–2H). The extent of reduction in ΔCm_peak_ is in the range, although on the lower side, of reported EPSC reduction by NSF inhibition in squid giant synapses and drosophila synapses.^2^^,^^3^^,^^4^ This difference might be due to differences in the stimulation protocol (depol_20ms_ vs. action potential), exocytosis detection methods (ΔCm vs. EPSC recordings), methods used to inhibit NSF, and the synapse under investigation.

A series of studies in the last two decades showed that calcium influx, but not the amount of exocytosis determined the rate of endocytosis, and a decrease in exocytosis alone does not slow down endocytosis.^8^^,^^9^^,^^10^^,^^12^^,^^13^ When a large amount of exocytosis saturates the endocytic capacity, a reduction of exocytosis will be accompanied by a decrease of endocytosis τ or an increased endocytosis rate.^8^^,^^14^^,^^15^^,^^16^^,^^17^ Thus, the decreased Rate_decay_n_ by NSF inhibition is not caused by the reduction of exocytosis itself, but by inhibition of endocytosis. Three sets of evidence below further support this suggestion. First, Rate_decay_n_ was normalized to the ΔCm_peak_, which normalized the contribution of the ΔCm_peak_ decrease on Rate_decay_. Second, ATPγS blocked endocytosis, but not ΔCm_peak_ or ICa within 4–10 min after break-in (Figures 1B, 1C, 2B, 2C, and S1), further confirming that endocytosis block can be independent of the ΔCm_peak_. At later dialysis time points, ATPγS reduced ΔCm_peak_ and ICa. We did not analyze these data because the ICa decrease may complicate analysis of the Rate_decay_n_.^9^ Third, at 2–4 min after whole-cell break-in, during which the exocytosis block was minimal, NSF_p_ and SNAP_p_ did not decrease the ΔCm_peak_, but still significantly reduced the Rate_decay_n_ induced by depol_20ms_ (Figures S2A–S2C) or depol_20msX10_ (Figures S2D–S2F).

### NSF and its SNARE disassembly activity are required for endocytosis at hippocampal synapses

The specificity of pharmacological blockers is a common concern in pharmacology experiments. We addressed this concern by (1) using four different NSF blockers in the calyx of Held, all of which generate the consensus inhibition of slow and fast endocytosis (Figures 1 and 2) and (2) knocking out or knocking down NSF genes as described below.

#### NSF conditional knockout mouse generation and gene deletion in culture synapses

**Figure 3:**
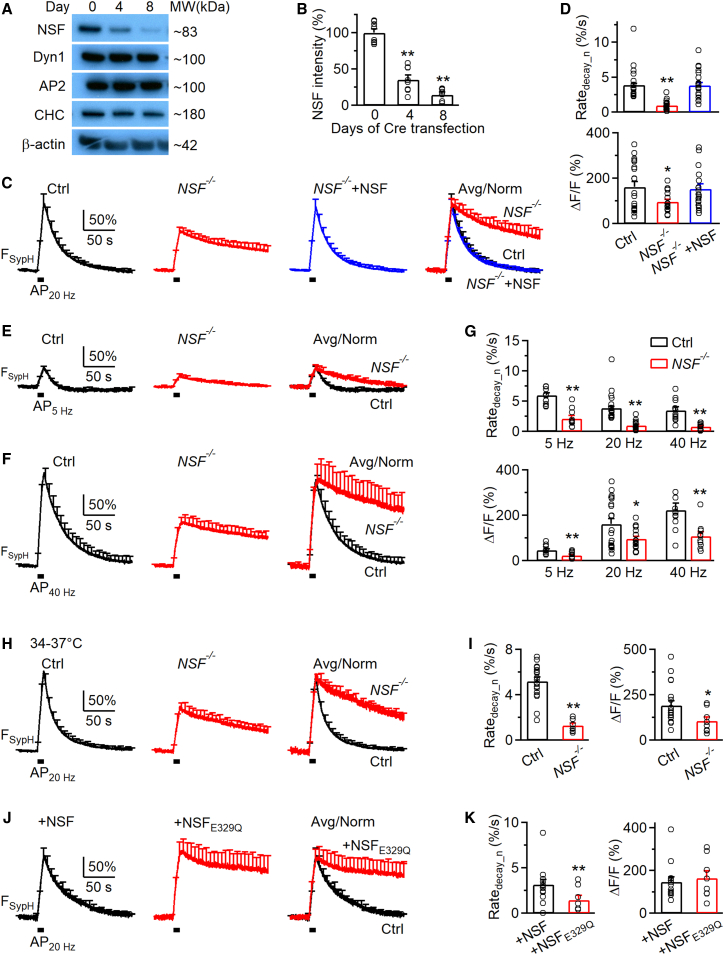
NSF knockout inhibits endocytosis at hippocampal synapses (A) Sampled western blot of NSF, clathrin heavy chain (CHC), adaptor protein 2 α subunit (AP2), dynamin 1 (Dyn 1), and β-actin at day 0, day 4, and day 8 after Cre-4-OHT treatment to the *NSF*^*LoxP/LoxP*^ hippocampal culture. (B) NSF intensity (mean + s.e.m.; 8 cultures) measured by western blot at days 0, 4, and 8 after Cre-4-OHT treatment in *NSF*^*LoxP/LoxP*^ hippocampal cultures. All data are normalized to NSF intensity at day 0. Each circle represents a single culture. (C) Averaged traces of SypH fluorescence (F_SypH_) changes induced by Train_20Hz_ in control (Ctrl, black, 20 experiments), *NSF*^−/−^ hippocampal boutons (red, 19 experiments), and *NSF*^−/−^ boutons overexpressed with wild-type NSF (*NSF*^−/−^ + NSF; blue; containing EBFP2 for recognition, 23 experiments) at 22°C–24°C. All data are expressed as mean + s.e.m. every 6 s (applies also to E, F, H, and J). Short bars under traces indicate the stimulation (applies also to E, F, H, and J). Traces are also normalized to control and overlapped to show the block of the F_SypH_ decay (right, Avg/Norm). Short black bars under traces indicate the stimulation (applies also to E, F, H, and J). Vertical scale bar, 50%, applies to the first three columns in (C); horizontal scale bar, 50 s, applies to all traces in (C). (D) Rate_decay_n_ (upper) and ΔF/F (lower) of SypH fluorescence (F_SypH_) shown in C (Ctrl, *NSF*^−/−^, and *NSF*^−/−^ + NSF). All data are expressed as mean + s.e.m. Each circle represents an experiment (applies also to G, I, and K). ∗*p* < 0.05; ∗∗*p* < 0.01 (*t* test). (E and F) The traces of F_SypH_ changes induced by a 10 s AP train at 5 Hz (E) or 40 Hz (F) in control (5 Hz: 11 experiments; 40 Hz: 11 experiments; black) and in *NSF*^−/−^ hippocampal boutons (5 Hz: 10 experiments; 40 Hz: 10 experiments; red) at 22°C–24°C. Traces are also normalized to control and overlapped to show the block of the F_SypH_ decay (right). Vertical scale bars, 50%, apply to the left and middle columns in (E) and (F); horizontal scale bars, 50 s, apply to all traces in (E) and (F). (G) Rate_decay_n_ (upper) and ΔF/F (lower) of F_SypH_ shown in (E) and (F). Data from (D) (AP_20Hz_) also included for comparison. ∗*p* < 0.05; ∗∗*p* < 0.01 (*t* test). (H and I) The traces (H), Rate_decay_n_ (I), and ΔF/F (I) of F_SypH_ changes induced by Train_20Hz_ in control (Ctrl, black, 19 experiments) and *NSF*^−/−^ hippocampal boutons (red, 9 experiments) at 34°C–37°C. Traces are also normalized and overlapped to show the block of the F_SypH_ decay (H, right). Vertical scale bar, 50%, applies to the left and middle columns in (H); horizontal scale bar, 50 s, applies to all traces in (H). ∗*p* < 0.05; ∗∗*p* < 0.01 (*t* test). (J and K) The traces (J), Rate_decay_n_ (K), and ΔF/F (K) of F_SypH_ changes induced by Train_20Hz_ in wild-type cultures expressed with wild-type NSF (+NSF, black, 14 experiments) or NSF_E329Q_ (+NSF_E329Q_, 8 experiments) at 22°C–24°C. Traces are also normalized to control and overlapped to show the block of the F_SypH_ decay (J, Avg/Norm, right). Vertical scale bar, 50%, applies to the left and middle columns in (J); horizontal scale bar, 50 s, applies to all traces in (J). ∗∗*p* < 0.01 (*t* test). See also Figures S3–S5.

To block NSF specifically, we generated NSF conditional knockout (*NSF*^*LoxP/LoxP*^) mice by floxing *NSF* exon 6 and exon 7 (Figure S3). In hippocampal neurons cultured from *NSF*^*LoxP/LoxP*^ mice, we deleted *NSF* by treating the culture with Cre-4-OHT or by Cre transfection (with mCherry for recognition).^18^ Western blot showed that Cre-4-OHT treatment progressively reduced NSF to below 20% in 8 days, but does not affect other endocytic proteins (dynamin, clathrin, and adaptor protein 2) (Figures 3A, 3B, and S4). Similar reduction was obtained at 8 days after Cre transfection, as detected with immunostaining (Figure S5). Since these two methods resulted in similar NSF reduction, we grouped them together as the *NSF*^−/−^ culture, and their corresponding data were grouped accordingly.

#### Synapto-pHluorin imaging of endocytosis

To record endocytosis, we transfected pH-sensitive synapto-pHluorin (SypH) to the *NSF*^−/−^ hippocampal culture and imaged SypH fluorescence (F_SypH_) from boutons at room temperature (22°C–24°C), if not mentioned otherwise.^12^^,^^14^ A train of action potential stimulation at 20 Hz for 10 s (AP_20Hz_) induced a F_SypH_ increase and decrease, reflecting exocytosis and endocytosis, respectively (Figure 3C). In control (*NSF*^*LoxP/LoxP*^ culture), the peak F_SypH_ increase over the baseline (ΔF/F) is 161.0 ± 23.4% (Figure 3D); F_SypH_ decay is mono-exponential with an initial decay rate (Rate_decay_n_) of 3.9 ± 0.2%/s (*n* = 20 experiments, each experiment contained ∼10–30 boutons; Figure 3D), where the rate was normalized to ΔF/F.

In *NSF*^−/*−*^ cultures, ΔF/F induced by AP_20Hz_ decreased ΔF/F to ∼60% of control, and reduced Rate_decay_n_ to about 25% of control (Figures 2C and 2D; *n* = 19 experiments). Both ΔF/F and Rate_decay_n_ were rescued to the control level by transfection of WT NSF to the *NSF*^−/*−*^ culture (Figures 3C and 3D; 20 experiments). These results suggest that NSF is required for mediating endocytosis after 20 Hz nerve firing.

Similar inhibition of both ΔF/F and Rate_decay_n_ was observed after 5 or 40 Hz action potential stimulation for 10 s (Figures 3E–3G), or AP_20Hz_ at physiological temperature (34°C–37°C; Figures 3H–3I). These results suggest that NSF is required for endocytosis regardless of the stimulation frequency and temperature.

#### ATP hydrolysis is required for endocytosis

The ATPase NSF hydrolyses ATP to disassemble the SNARE complex, which was blocked by the NSF E329Q mutant (NSF_E329Q_).^19^^,^^20^ To determine whether NSF’s ATPase function in disassembling the SNARE complex is needed for endocytosis, we first overexpressed NSF_E329Q_ in the *NSF*^−/*−*^ culture, but found that imaging was not possible because most neurons were dead for an unknown reason. We then overexpressed either NSF (used as the control) or NSF_E329Q_ in wild-type hippocampal neurons. We found that AP_20Hz_-induced Rate_decay_n_ was ∼3.3%/s with NSF transfection (control), but was reduced to ∼44% of control with NSF_E329Q_ transfection (Figures 3J and 3K), suggesting that ATPase-mediated SNARE complex disassembly is required for endocytosis at hippocampal synapses.

### NSF involvement in bulk endocytosis at hippocampal synapses observed with EM

**Figure 4:**
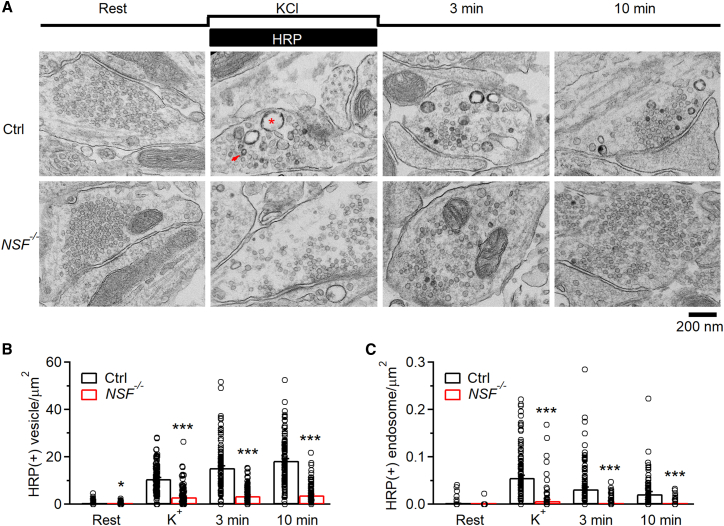
NSF knockout affects endocytosis examined with EM at hippocampal synapses (A) EM images of WT and *NSF*^−/−^ hippocampal boutons at rest (Rest) and at 0 min (KCl), 3 min, and 10 min after 1.5 min 90 mM KCl application. For Rest, HRP was included for 1.5 min; for KCl application, HRP was included only during KCl application (see labels). ∗: an HRP(+) bulk endosome; arrow: an HRP(+) vesicle. Horizontal scale bar (200 nm) applies to all images in (A). (B and C) Number of HRP(+) vesicles (B) and the bulk endosome area (C) per square micrometer of synaptic cross-section are plotted versus the time before (Rest) and at 0 min (KCl), 3 min, and 10 min after the end of KCl application in WT and *NSF*^−/−^ hippocampal cultures. Data are expressed as mean + s.e.m. Each group comprised 120–122 synaptic profiles from 18 mice (3 male and 3 female mice per experiment, 3 independent experiments). The temperature before fixation was 37°C. ∗*p* < 0.05; ∗∗∗*p* < 0.001 (*t* test). Each circle represents one synaptic profile. See also Figures S3 and S5.

We performed EM to examine the ultrastructural changes in *NSF*^−/−^ hippocampal cultures at physiological temperature. Horseradish peroxidase (HRP, 5 mg/mL) was added in bath for assay of vesicular uptake. At rest, HRP-positive [HRP(+)] vesicles were minimal; most vesicles were HRP-negative [HRP(−)] (Figure 4A); the number of HRP(+) vesicles in boutons was similar in Ctrl and *NSF*^−/−^ cultures. To examine endocytosis, we applied 90 mM KCl with HRP for 1.5 min, and fixed samples at 0, 3, and 10 min after KCl/HRP application. In Ctrl boutons, compared with the resting condition, HRP(+) vesicles increased from time 0 to 10 min after KCl, reflecting vesicle endocytosis (Figures 4A and 4B) as previously shown.^21^^,^^22^ Compared with Ctrl boutons, HRP(+) vesicles were significantly reduced at each time point after KCl application in *NSF*^−/−^ boutons (Figures 4A and 4B), suggesting inhibition of endocytosis of regular vesicles.

In Ctrl boutons, we observed HRP(+) bulk endosomes (Figure 4A), defined as vesicles with a diameter 80 nm or with a cross-section area more than that of an 80 nm vesicle (∼0.005 μm^2^). Bulk endosome area increased at time 0, then decreased at 3 and 10 min (Figures 4A–4C), suggesting generation of bulk endosomes and subsequent conversion to vesicles as previously shown.^10^^,^^21^ Similar trends were observed in *NSF*^−/−^ cultures, but at a significantly lower level (Figures 4A–4C), suggesting inhibition of bulk endocytosis. Thus, EM results reveal the involvement of NSF in regular vesicle endocytosis and bulk endocytosis at hippocampal synapses.

### NSF is essential for pore closure of preformed and fusion-generated Ω-profiles in chromaffin cells

We showed NSF involvement in slow, fast, and bulk endocytosis at synapses (Figures 1, 2, 3, and 4). Since these different endocytic modes are mediated primarily by the pore closure of preformed Ω-profiles (pre-Ω, formed before depolarization) and fusion-generated Ω-profiles (fs-Ω) in chromaffin cells,^23^^,^^24^^,^^25^ we determined whether NSF involvement in endocytosis is due to its role in closing pre-Ω and fs-Ω’s pore in chromaffin cells in the following.

#### Methods for imaging pre-Ω and fs-Ω pore closure

**Figure 5:**
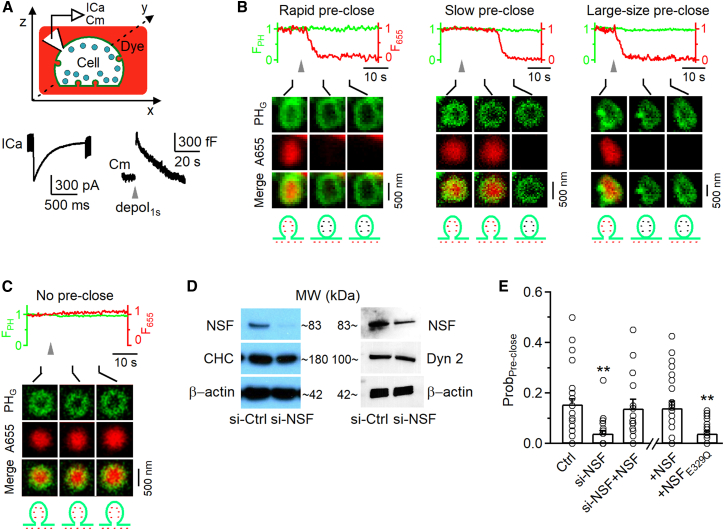
NSF is essential for mediating pre-Ω pore closure in chromaffin cells (A) Upper: setup drawing. The cell membrane, bath, and vesicles are labeled with PH_G_ (green), A655 (red), and FFN511 (blue), respectively. ICa and Cm are recorded via a whole-cell pipette. Lower: Sampled ICa (left) and Cm (right) changes induced by depol_1s_ (gray arrowhead). Left: vertical scale bar, 300 pA; horizontal scale bar, 500 ms. Right: vertical scale bar, 300 fF; horizontal scale bar, 20 s. (B) PH_G_ fluorescence (F_PH_), A655 fluorescence (F_655_) and sampled confocal images showing depol_1s_-induced rapid (left), slow (middle) or large-size (right) pre-spot pore closure (pre-close) in chromaffin cells. F_PH_ and F_655_ were normalized to the baseline. Labels for two *y* axes apply to the other images in (B). Gray arrowheads indicate a 1-s stimulation. Schematic cartoons in the bottom image illustrate the process of vesicle closure. Upper horizontal scale bars, 10 s; lower vertical scale bars, 500 nm. (C) F_PH_, F_655_, and sampled confocal images showing a pre-spot without undergoing pore closure after depol_1s_ in a chromaffin cell (no pre-close). F_PH_ and F_655_ were normalized to the baseline. Gray arrowhead indicates a 1-s stimulation. Upper horizontal scale bar, 10 s; lower vertical scale bar, 500 nm. (D) Western blot of NSF, clathrin heavy chain (CHC), dynamin 2 (Dyn 2), and β-actin in chromaffin cell cultures transfected with si-Ctrl or si-NSF. (E) The probability of pre-spots undergoing pre-close after depol_1s_ (Prob_pre-close_) in control (Ctrl, 25 cells), si-NSF transfection (22 cells), si-NSF transfection plus wild-type NSF overexpression (si-NSF+NSF, 15 cells), control cells overexpressed with wild-type NSF (+NSF, 22 cells), or control cells overexpressed with NSF_E329Q_ (+NSF_E329Q_, 24 cells). Data are expressed as mean + s.e.m. Each circle presents the data from a cell. ∗∗*p* < 0.01 (*t* test, compared to control). See also Figures S6 and S7.

**Figure 6:**
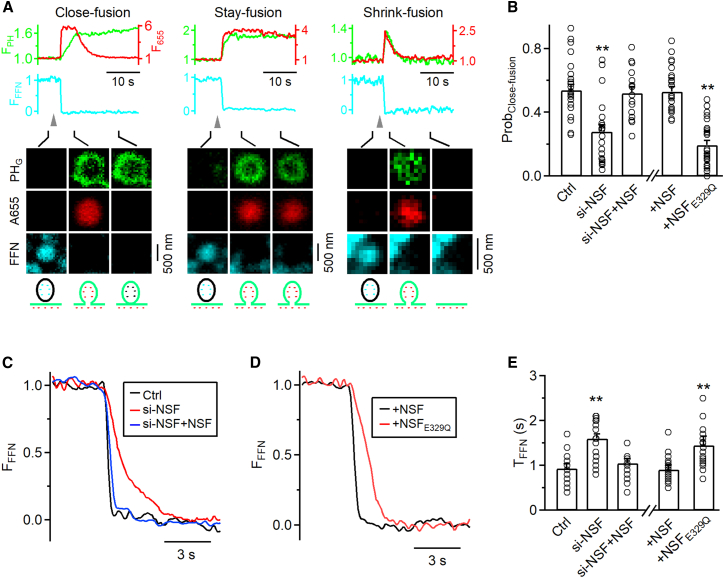
NSF in mediating fusion (fs-Ω) pore closure and facilitating content release in chromaffin cells (A) PH_G_ fluorescence (F_PH_, green), A655 fluorescence (F_655_, red), FFN511 fluorescence (F_FFN_, blue), and confocal images showing close- (left), stay- (middle) and shrink-fusion (right). Labels for three *y* axes apply to the other images in (A). Gray arrowheads indicate a 1-s stimulation. Schematic cartoons in the bottom image illustrate the process of vesicle fusion. Upper horizontal scale bars, 10 s. Lower vertical scale bars, 500 nm. (B) The probability for a fusion spot to undergo close-fusion (Prob_close-fusion_) measured after depol_1s_ in control (Ctrl, 25 cells), cells transfected with si-NSF (22 cells), cells transfected with si-NSF transfection plus wild-type NSF overexpression (si-NSF+NSF, 15 cells), control cells overexpressed with wild-type NSF (+NSF, 22 cells), or control cells overexpressed with NSF_E329Q_ (+NSF_E329Q_, 24 cells). Data are expressed as mean + s.e.m. Each circle represents data from a cell. ∗∗*p* < 0.01 (*t* test, compared to control). (C) Averaged FFN511 spot fluorescence (F_FFN_) decay due to fusion in three groups: control (Ctrl, 20 fusion events from 4 cells), si-NSF (20 fusion events from 4 cells), and si-NSF+NSF (19 fusion events from 4 cells). Horizontal scale bar, 3 s. (D) Averaged FFN511 spot fluorescence (F_FFN_) decay due to fusion in control cells overexpressing wild-type NSF (+NSF, 20 fusion events from 4 cells) or overexpressing NSF_E329Q_ (+NSF_E329Q_, 24 fusion events from 4 cells). Horizontal scale bar, 3 s. (E) The 20%–80% decay time (T_FFN_) of F_FFN_ (indicating release time) in Ctrl (25 cells), si-NSF (22 cells), si-NSF+NSF (15 cells), +NSF (22 cells), +NSF_E329Q_ (24 cells). Data are expressed as mean + s.e.m. Each circle represents data from a cell. ∗∗*p* < 0.01 (*t* test, compared to control). See also Figures S6 and S7.

We have developed imaging methods to detect pre-Ω (Figure 5) and fs-Ω (Figure 6) pore closure in live adrenal chromaffin cells.^23^^,^^24^^,^^25^^,^^26^^,^^27^ Pre-Ω (∼200–1,500 nm in diameter; Figure 5B) could be generated from (1) the endocytic flat-to-Ω-shape transition, including bulk endocytosis that produces vesicles larger than fusing vesicles^25^^,^^28^ and (2) dense-core vesicle fusion, some of which could maintain the Ω-shape for a long time.^25^ Fs-Ω is from fusion of dense-core vesicles^24^ with a diameter of ∼360 nm (range: 200–700 nm, Figure 6A).^29^^,^^30^ We used mNeonGreen attached to phospholipase C δPH domain (PH_G_, overexpressed, binds to PI(4,5)P_2_) to label the plasma membrane (PM), Atto 655 (A655, 30 μM in bath; or Atto 532) to fill Ω-profiles, and fluorescent false neurotransmitter FFN511 (or FFN206) pre-loaded into vesicles to measure release (Figures 5B, 6A, and S6).^18^^,^^25^ At the bottom plasma membrane of resting cells, XY-plane confocal microscopy observed FFN511-containing vesicle spots and preformed PH_G_ spots and rings overlapped with A655, but not FFN511 spots (termed pre-spot; Figures 5B and S6). Pre-spots were mostly pre-Ω as observed at the XZ-plane with confocal or stimulated emission depletion (STED) microscopy (e.g., Figure S6, for detail, see Shin et al.^25^).

A whole-cell 1-s depolarization (−80 to +10 mV, depol_1s_) induced ICa, capacitance changes reflecting exo-endocytosis (Figure 5A), pre-spot closure (pre-close; Figure 5B), and fusion spots observed with confocal microscopy (Figure 6A; cell-bottom, XY-plane imaging every 40–80 ms).^23^^,^^24^^,^^25^ Pre-close was detected as A655 fluorescence (F_655_, strongly excited) dimming while PH_G_ fluorescence (F_PH_, weakly excited) sustained or dimmed with a delay (Figure 5B, see Figure 5C for a pre-spot without undergoing pore closure).^25^ This method detected pore closure of pre-Ω that was impermeable to H^+^ and OH^−^, mediated by dynamin, and observed directly with STED imaging.^23^^,^^24^^,^^25^^,^^26^ Pre-Ω closure forms ∼200–1,500 nm vesicles,^25^ with ∼17% in the 600–1,500 nm range (e.g., Figure 5B, right) that can be attributed to bulk endocytosis.^25^

Fusion spots were detected as a sudden appearance of PH_G_ and A655 spots while FFN511 spot fluorescence (F_FFN_) decayed, due to the diffusion of PH_G_/A655 from the PM/bath to the fs-Ω and release of FFN511 from the fs-Ω (Figure 6A). Three fusion modes were observed (see “STAR Methods” for more detail): (1) close-fusion (kiss-and-run)—fs-Ω pore closure was detected similarly to pre-close: as F_655_ dimming while F_PH_ was sustained or decayed later (Figure 6A, left)^23^^,^^24^^,^^25^^,^^26^; (2) stay-fusion—a sustained fs-Ω was detected as persistent PH_G_/A655 spots with sustained F_655_ and F_PH_ (Figure 6A, middle); (3) shrink-fusion—fs-Ω shrinking was detected as parallel decreases of spot-size with F_655_ and F_PH_ (Figure 6A, right).^24^^,^^26^^,^^31^ STED imaging directly observed these modes (for detail, see Shin et al.^24^^,^^31^).

#### NSF and its ATPase activity are required for pre-Ω and fs-Ω pore closure

NSF siRNA (si-NSF) transfection substantially reduced NSF without affecting key endocytic protein dynamin and clathrin (Figures 5D and S7). si-NSF substantially reduced the probability of pre-spots to undergo pre-close (pore closure) induced by depol_1s_ (Prob_pre-close_; Figure 5E) and the probability of fusion spots to undergo close-fusion (Prob_close-fusion_; Figure 6B), but increased the FFN511 20%–80% decay time that reflects the release time course (T_FFN_-release time; Figures 6C–6E). NSF overexpression in si-NSF-transfected cells rescued pre-close (Figure 5E), close-fusion (Figure 6B), and T_FFN_ (Figures 6C–6E) to the control level. These results suggest that NSF is required for pre-close and close-fusion, and controls the time course of vesicular content release.

Overexpression of NSFE_329Q_, a mutant unable to disassemble SNARE complexes,^19^^,^^20^ reduced Prob_pre-close_ (Figure 5E) and Prob_close-fusion_ (Figure 6B) measured after depol_1s_, but increased T_FFN_ (Figures 6D and 6E), suggesting that SNARE disassembly by NSF is required for closing both pre-Ω and fs-Ω’s pore, and controlling content release time course.

### NSF mediates slow, fast, ultrafast, and overshoot endocytosis by closing pre-Ω/fs-Ω’s pore in chromaffin cells

**Figure 7:**
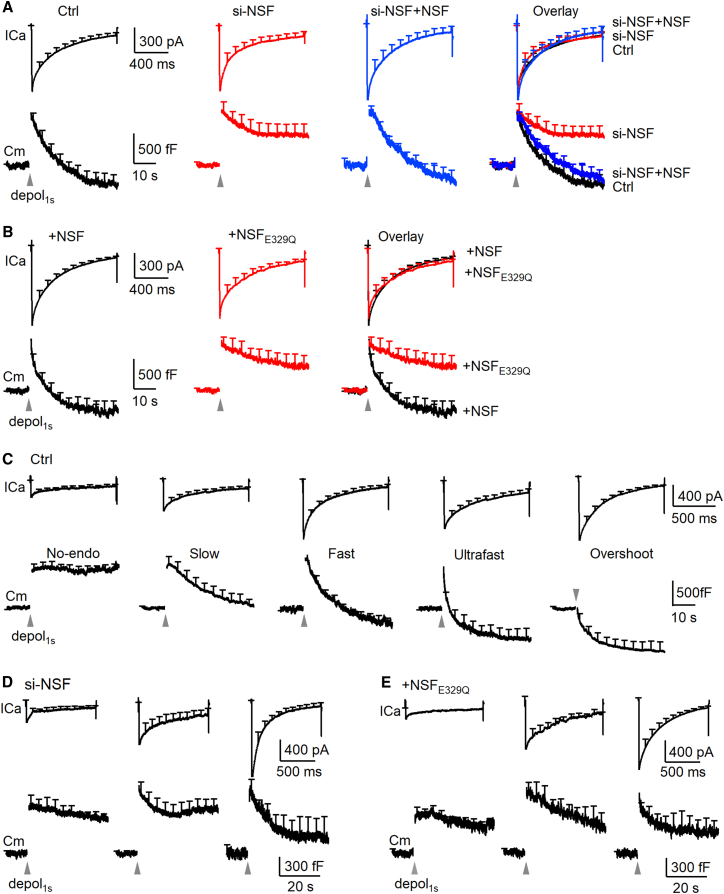
NSF is essential for mediating slow, fast, ultrafast, and overshoot endocytosis in chromaffin cells (A) Depol_1s_-induced ICa (upper) and Cm (lower) in chromaffin cells under control (Ctrl, black, 25 cells), si-NSF transfection (si-NSF, red, 22 cells), and si-NSF transfection plus wild-type NSF overexpression (si-NSF+NSF, blue, 15 cells). Data are expressed as mean + s.e.m. at intervals of 0.1 s (ICa) or 4 s (Cm); gray arrowheads indicate a 1-s stimulation (applies to all other images in this figure). Traces are also merged on the right for comparison. Upper: vertical scale bar, 300 pA; horizontal scale bar, 400 ms. Lower: vertical scale bar, 500 fF; horizontal scale bar, 10 s. The scale bars apply to all images in (A). (B) Depol_1s_-induced ICa (upper) and Cm (lower) in chromaffin cells overexpressed with wild-type NSF (+NSF, black, 22 cells) or NSF_E329Q_ (+NSF_E329Q_, red, 24 cells). Traces are also merged in the right. Upper: vertical scale bar, 300 pA; horizontal scale bar, 400 ms. Lower: vertical scale bar, 500 fF; horizontal scale bar, 10 s. The scale bars apply to all images in (B). (C) Mean ICa (upper) and Cm (lower) induced by depol_1s_ in five groups of chromaffin cells (from left to right): Group_no-endo_ (decay <30% ΔCm, 6 cells), Group_slow_ (endocytic τ > 6 s, 6 cells), Group_fast_ (τ: 0.6–6 s, 5 cells), Group_ultrafast_ (τ < 0.6 s, 4 cells) and Group_overshoot_ (decay >130% ΔCm, 4 cells) in control chromaffin cells. Upper: vertical scale bar, 400 pA; horizontal scale bar, 500 ms. Lower: vertical scale bar, 500 fF; horizontal scale bar, 10 s. The scale bars apply to all images in (C). (D and E) Depol_1s_-induced ICa (upper) and Cm (lower) in chromaffin cells with ICa of 160–360 pA (left), 400–900 pA (middle), and 1,000–1,800 pA (right) in two conditions: (D) si-NSF transfection (left: 8 cells; middle: 7 cells; right: 7 cells); (E) NSF_E329Q_ overexpression (left: 9 cells; middle: 8 cells; right: 7 cells). Upper: vertical scale bars, 400 pA; horizontal scale bars, 500 ms. Lower: vertical scale bars, 300 fF; horizontal scale bars, 20 s. The scale bars apply to all images in (D) and (E).

Since endocytosis in chromaffin cells is primarily due to pre-Ω/fs-Ω’s pore closure rather than the endocytic transformation,^18^^,^^25^ our finding of NSF involvement in pre-Ω/fs-Ω pore closure predicts NSF involvement in endocytosis. We verified this prediction by examining how inhibition of NSF affects depol_1s_-induced capacitance decay (after the jump) that reflects endocytosis. The following two sets of results suggest that NSF and its SNARE-disassembly function are required for endocytosis in chromaffin cells. First, si-NSF transfection inhibited the Cm-decay-indicated endocytosis, but not ICa; and the inhibition was rescued by overexpression of wild-type NSF (Figure 7A). Second, overexpression of NSFE_329Q_ inhibited the Cm-decay-indicated endocytosis as compared to overexpression of wild-type NSF (Figure 7B).

To determine which mode(s) of endocytosis NSF is involved in, we first described five distinct modes of endocytosis with different time constants and amplitudes as recently characterized systematically in chromaffin cells.^18^^,^^25^ Five modes of endocytosis were revealed when chromaffin cells were divided into five groups based on the decay of the whole-cell capacitance (Cm) after the jump induced by depol_1s_: (1) no-endocytosis (Group_no-endo_, decay < 30% ΔCm), (2) slow endocytosis (Group_slow_, endocytic τ > 6 s), (3) fast endocytosis (Group_fast_, τ: 0.6–6 s), (4) ultrafast endocytosis (Group_ultrafast_, τ < 0.6 s), and 5) overshoot endocytosis (Group_overshoot_, decay > 130% ΔCm; Figure 7C; see Shin et al.^25^ for detail). Calcium influx triggers endocytosis in each of these five groups: larger ICa induces faster and larger amplitude of endocytosis (Figure 7C), whereas strontium abolishes endocytosis (see Shin et al.^25^ for detail).

si-NSF transfection or NSF_E329Q_ overexpression inhibited the Cm-decay-indicated endocytosis in all three cell groups divided based on the ICa amplitude (Figures 7D and 7E), indicating inhibition of endocytosis throughout the entire ICa range that generates five distinct endocytic modes in control (Figure 7C). These results suggest that NSF and its SNARE complex disassembly function are required to drive slow, fast, ultrafast, and overshoot endocytosis observed in control. We concluded that NSF and its SNARE complex disassembly function contribute to mediating diverse modes of endocytosis by participating in closing the pre-Ω and fs-Ω’s pore.

## Discussion

While finding the involvement of the core “exocytosis” protein NSF in endocytosis is apparently surprising, it is consistent with a series of studies showing that other core exocytosis proteins, including SNAP-25, syntaxin, VAMP2, and synaptotagmin 1, are involved in synaptic vesicle endocytosis at calyx-type and hippocampal synapses.^13^^,^^32^^,^^33^^,^^34^^,^^35^^,^^36^^,^^37^ It may also provide an explanation for an early study showing that NSF inhibitors reduced the total vesicle number in squid giant nerve terminals^3^—the block of endocytosis reduces the vesicle supply. The dual roles of these core “exocytosis” proteins in both exo- and endocytosis suggest that they may play an important role in coupling exocytosis to endocytosis, which recycles vesicles and maintains the exocytosis capacity and membrane homeostasis of release sites.^27^^,^^38^

In line with its dual roles in exo- and endocytosis, we demonstrated that NSF and its SNARE-disassembly function are required to close the pore of fs-Ω (fusion pore) and pre-Ω, thereby mediating endocytosis in chromaffin cells (Figures 5 and 6). Given that pre-Ω could be generated by fusing vesicles that maintain a Ω-shape, NSF and its SNARE-disassembly function may mediate fusion pore closure and thus endocytosis via the kiss-and-run (close-fusion) and kiss-and-stay fusion (stay-fusion). Since pre-Ω could also be generated from endocytic flat-to-Ω shape transition,^25^^,^^39^ NSF and its SNARE-disassembly function may be required for pore closure during classical endocytic flat-to-round vesicle formation.

How is NSF involved in pore closure? A recent study shows that SNARE disassembly by NSF is essential for closing the fusion pore formed in the *in vitro* SNARE-reconstituted nanodisk.^40^ The SNARE complex formation is required to assemble a fusion pore, whereas the SNARE complex disassembly by NSF has been suggested to disassemble the fusion pore, resulting in the fusion pore closure.^40^ This *in vitro* finding offers a mechanistic explanation for why NSF and its SNARE-disassembly function are required to mediate fusion pore closure in live chromaffin cells. An alternative explanation could be that NSF-mediated SNARE disassembly may help to clear the release site,^9^^,^^33^^,^^38^^,^^41^ allowing for the endocytic proteins, such as dynamin^23^^,^^24^ and the recently found clathrin,^18^ to access and close the fusion pore.

This explanation seems difficult to account for the requirement of NSF and its SNARE-complex disassembly function in closing the pre-Ω’s pore. It might be possible that after fusion, the SNARE complex is sorted to the endocytic site around the pre-Ω’s pore region, which may present a physical barrier that may prevent dynamin and clathrin from accessing the pore. Disassembly of the SNARE complex at the pre-Ω by NSF might thus facilitate pre-Ω pore closure, explaining why NSF is needed for the pre-Ω pore closure. While beyond the scope of the present work, it is interesting to determine how NSF is involved in pore closure in the future.

Our findings provide an explanation for a long-standing observation that endocytosis requires energies from not only GTP hydrolysis,^17^^,^^42^ but also ATP hydrolysis^43^ —NSF hydrolyzes ATP to disassemble the SNARE complex. Through its ATPase activity that disassembles the SNARE complex, NSF (1) mediates diverse modes of endocytosis, as shown here, (2) regulates the trafficking of neurotransmitter receptors, including AMPA receptors, GABA receptors, and dopamine receptors, and (3) contributes to generating synaptic plasticity in the nervous system across different types of synapses.^6^ The dysfunction of NSF-mediated trafficking of these receptors or NSF mutations is associated with several neurological disorders, such as Alzheimer’s disease and epilepsy.^6^ Our finding that NSF mediates diverse endocytic modes by closing pre-Ω and fs-Ω’s pore provides a mechanistic basis accounting for these physiological and pathological roles of NSF.

### Limitations of the study

NSF E329Q mutation was reported to inhibit SNARE complex disassembly.^19^^,^^20^ We could not exclude its potential for other non-specific effects, as we were unable to measure SNARE complex disassembly and thus verify its inhibitory role in chromaffin cells or hippocampal synapses. However, its inhibitory effect on endocytosis is consistent with the effects of peptides we used to inhibit NSF’s function in disassembling the SNARE complex in the calyx of Held (Figures 1 and 2), supporting NSF E329Q mutation in inhibiting SNARE complex disassembly.

While we have demonstrated clearly a crucial function of NSF in endocytosis via pre-Ω and fs-Ω pore closure, we have not explored its underlying mechanisms. It would be of great interest to explore underlying mechanisms in the future.

## Resource availability

### Lead contact

Further information and requests for resources and reagents should be directed to and will be fulfilled by the lead contact, Ling-Gang Wu (wul@ninds.nih.gov).

### Materials availability

This study did not generate new unique reagents.

### Data and code availability

All data produced for this manuscript are available from the lead contact (wul@ninds.nih.gov) upon reasonable request. This paper does not report original code. Any additional information required to reanalyze the data reported in this paper is available from the corresponding author upon request.

## Acknowledgments

We thank Jianhua Xu for the strong support of calyx experiments, Susan Cheng and Virginia Crocker for EM technical support, Dr. Gero Miesenböck (University of Oxford, Oxford, UK) for providing us with the synapto-pHluorin plasmid, and Dr. Yongling Zhu for synaptophysin-pHluorin2X plasmid. This work was supported by 10.13039/100000065NINDS Research Program (ZIA NS003009-15 and ZIA NS003105-10 to L.G.W.). The contributions of the NIH author(s) were made as part of their official duties as NIH federal employees, are in compliance with agency policy requirements, and are considered Works of the United States Government. However, the findings and conclusions presented in this paper are those of the author(s) and do not necessarily reflect the views of the NIH or the 10.13039/100000016U.S. Department of Health and Human Services.

## Author contributions

L.G.W., conceptualization, supervision, writing – review and editing, and funding acquisition; X.S.W., T.S., and B.S., methodology, validation, formal analysis, visualization, and writing – original draft; X.S.W., T.S., and B.S., investigation (major experiments); Z.Z., S.L., L.W., X.W., M.M., S.H., and L.G., investigation (minor experiments); S.L., validation and formal analysis. All authors have read and agreed to the published version of the article.

## Declaration of interests

All authors declare no competing interests.

## STAR★Methods

### Key resources table

**Table:** 

REAGENT or RESOURCE	SOURCE	IDENTIFIER
**Antibodies**		

Mouse monoclonal anti-β-actin	Abcam	Cat#ab6276; RRID: AB_2223210
Rabbit polyclonal anti-AP2	Thermo Fisher Scientific	Cat#PA1-41068; RRID: AB_2115072
Rabbit polyclonal anti-CHC (Clathrin heavy chain)	Abcam	Cat#ab21679; RRID: AB_2083165
Mouse Monoclonal anti-dynamin 1	Cell Signaling Technology	Cat#4565; RRID: AB_2093212
Rabbit Monoclonal anti-dynamin 2	Cell Signaling Technology	Cat# 90438; RRID: AB_3739825
Mouse monoclonal anti-NSF-1	Abcam	Cat#ab16681; RRID: AB_2155806
Rabbit monoclonal anti-TAU	Abcam	Cat#ab32057; RRID: AB_778254

**Biological samples**		

Bovine adrenal glands	J. W. Treuth & Sons Inc.	https://www.jwtreuth.com

**Chemicals, peptides, and recombinant proteins**		

Atto 655 carboxy	ATTO-TEC	Cat#AD655-21
FFN511	Abcam	Cat#ab-120331
Horseradish peroxidase (HRP)	Sigma-Aldrich	Cat#P8125
Lipofectamine™ LTX Reagent with PLUS™ Reagent	ThermoFisher Scientific	Cat#15338030
Mutated NSF peptide (NSF_mp_): TGKTLIARKIETMLNAREPK	21st Century Biochemicals	N/A
NSF peptide (NSF_p_): TGKTLIARKIGTMLNAREPK	21st Century Biochemicals	N/A
SNAP peptide (SNAP_p_): QSFFSGLFGGSSKIEEACE	21st Century Biochemicals	N/A
Scrambled SNAP peptide (SNAP_sp_): GFAESLFQSIEKESGFSCG	21st Century Biochemicals	N/A
Tetrodotoxin (TTX)	Tocris	Cat#1078

**Critical commercial assays**		

Elite ABC-HRP kit	Vector Labs	Cat#PK-6100
Basic Nucleofector^TM^ Kit for primary mammalian neurons	Lonza	Cat#VPI-1003
Quick extract DNA extraction solution	Lucigen	Cat#QE09050

**Experimental models: Organisms/strains**		

Mouse: C57BL/6J	Jackson Laboratory	Cat#000664; RRID:IMSR_JAX: 000664
Mouse: *NSF*^*LoxP*^	Lin Gan’s lab (This paper)	N/A
Rat: Wistar	Charles River Laboratories	Cat#003

**Oligonucleotides**		

MISSION® esiRNA (siRNA NSF)	Sigma-Aldrich	Cat#EHU051331
MISSION® siRNA Fluorescent Universal Negative Control #1, Cyanine 3 (si-Control)	Sigma-Aldrich	Cat#SIC003

**Recombinant DNA**		

Plasmid: cDNA NSF (Mouse)	NovoPro	N/A
Plasmid: cDNA NSF_E329Q_ (Mouse)	Genecopoeia	N/A
Plasmid: pH-sensitive pHluorin 2X (SypH)	Yong-Ling Zhu’s lab (Zhu et al.^44^)	N/A

**Software and algorithms**		

Igor Pro 6.1.2.1	WaveMetrics, Inc.	https://www.wavemetrics.com
Image J	National Institutes of Health	https://imagej.net/
NIS-Elements AR 4.1	Nikon	NIS-Elements | Software | Microscope Products | Nikon Instruments Inc.
Pulse v8.67	HEKA Elektronik	https://www.heka.com

**Other**		

EPC-10 Amplifier	HEKA Elektronik	RRID: SCR_018399
German glass coverslips with mouse laminin coating over PDL layer	Neuvitro	Cat#GG-25-Laminin
Integraslice 7550 Vibratome	Campden Instruments	https://www.campdeninstruments.com
JEOL200CX transmission electron microscope	JEOL	https://www.jeolusa.com
Nikon Eclipse A1 confocal microscope	Nikon	https://www.microscope.healthcare.nikon.com

### Experimental model and study participant details

#### Animal care and use

All animal procedures were performed in accordance with NIH guidelines and were approved by the NIH Animal Care and Use Committee (NINDS ASP-1170 and ASP-1259). Wistar rats were obtained from Charles River Laboratories, and wild-type C57BL/6J mice were obtained from The Jackson Laboratory. *NSF*^*loxP*^ mice were generated by Dr. Lin Gan and described in Figure S3. *NSF*^*loxP/loxP*^ mice of either sex were obtained by heterozygous and homozygous breeding using standard mouse husbandry procedures. Mouse genotypes were determined by PCR. Rats and mice were housed under controlled environmental conditions: temperature 70–74°F, humidity 35–60%, with a 12 h light/12 h dark cycle (light: 6 AM–6 PM, dark: 6 PM–6 AM).

Fresh bovine adrenal glands, obtained from male and female bovines aged 21–27 months, were purchased from J. W. Treuth & Sons, Inc.

P7–10 rats of either sex were used for the preparation of brainstem slices. P0 mice of either sex were used for hippocampal culture experiments. The adrenal glands were used for chromaffin cell culture experiments.

### Method details

#### Slice preparation, capacitance recordings and solutions

Slice preparation and capacitance recordings were similar as previously described.^9^^,^^15^^,^^45^^,^^46^^,^^47^ Briefly, parasagittal brainstem slices (200 μm thick) containing the medial nucleus of the trapezoid body were prepared from 7–10-day-old male or female Wistar rats using a vibratome. Whole-cell membrane capacitance measurements were performed using an EPC-10 amplifier in combination with a software lock-in amplifier (PULSE; HEKA, Lambrecht, Germany) implementing the Lindau-Neher technique. The frequency of the sinusoidal stimulus was 1,000 Hz, and the peak-to-peak voltage of the sine wave was ≤ 50 mV. We pharmacologically isolated presynaptic Ca^2+^ currents with a bath solution (∼22–24°C) containing (in mM): 105 NaCl, 20 TEA-Cl, 2.5 KCl, 1 MgCl_2_, 2 CaCl_2_, 25 NaHCO_3_, 1.25 NaH_2_PO_4_, 25 glucose, 0.4 ascorbic acid, 3 *myo*-inositol, 2 sodium pyruvate, 0.001 tetrodotoxin (TTX), 0.1 3,4-diaminopyridine, 300–310 mOsm, pH 7.4 when bubbled with 95% O_2_ and 5% CO_2_. The presynaptic pipette contained (in mM): 125 Cs-gluconate, 20 CsCl, 4 MgATP, 10 Na_2_-phosphocreatine, 0.3 GTP, 10 HEPES, 0.05 BAPTA, 310–320 mOsm, pH 7.2, adjusted with CsOH.

NSF peptide (TGKTLIARKIGTMLNAREPK), mutated NSF peptide (TGKTLIARKIETMLNAREPK), SNAP peptide (QSFFSGLFGGSSKIEEACE), scrambled SNAP peptide (GFAESLFQSIEKESGFSCG) were purchased from the 21st Century Biochemicals, Inc. (Marlboro, MA, USA).

#### Measurements of the time constant, Rate_decay_ and Rate_decay_n_ in calyces

The τ was measured from exponential fit of Igor (Figures 1B and 2B). Rate_decay_ at calyces was measured between 0.5–4 s after depol_20ms_ that induced slow endocytosis, but between 0.5–1.5 s after depol_20msX10_ that induced rapid endocytosis.^9^^,^^10^^,^^11^ Rate_decay**_**n_ was measured as Rate_decay_ divided by the capacitance jump peak amplitude (ΔCm_peak_). We used depol_20msX10_ to induce rapid endocytosis, because the Rate_decay_ after depol_20msX10_ reflected mostly (∼80%) the rapid component of endocytosis.^9^^,^^10^^,^^12^

#### Mouse hippocampal culture and transfection

Mouse hippocampal culture was prepared as described previously.^10^^,^^48^ Hippocampal CA1-CA3 regions were dissected from P0–P1 wild-type or *NSF*^*Loxp/Loxp*^ mice of either sex, dissociated, and plated on Poly-D-lysine. Cells were maintained at 37°C in a 5% CO_2_ humidified incubator in a medium containing MEM, 0.5% glucose, 0.1 g/l bovine transferrin, 0.3 g/l glutamine, 10% fetal bovine serum, 2% B-27, and 3 μM cytosine β-D-arabinofuranoside. On 6–8 days after plating, neurons were transfected with plasmids using Lipofectamine LTX. Neurons were then maintained at 37°C for an additional 2 days before imaging.

Transfected plasmids included a plasmid containing synaptophysin-pHluroin (SypH) gifted by Dr. Yongling Zhu^44^ alone (control) or with a L309 plasmid containing Cre/mCherry. A nuclear localization sequence was tagged at the N-terminal of Cre, and cloned into L309 vector via BamHI and EcoRI sites. Accordingly, mCherry was expressed in the nucleus. For the rescue experiments (see Figure 2), we transfected cDNA NSF plasmid (Novopro) along with SypH and Cre/mCherry. The cDNA encoding NSF was subcloned into EBFP2-C1 (Addgene #54665), and EBFP2 was used for us to recognize transfected cells.

The cDNA encoding NSF_E329Q_ (Genecopoeia) was subcloned into EBFP2-C1 (Addgene #54665) and EBFP2 was used for us to recognize transfected cells. For the rescue experiments, we transfected NSF plasmid along with SypH and NSF_E329Q_.

#### Immunohistochemistry in hippocampal cultures

Cells were fixed with 4% paraformaldehyde, permeabilized with 0.3% Triton X-100, and subsequently incubated with primary and secondary antibodies. Primary antibodies were diluted in PBS containing 10% donkey serum and incubated with cells at 4^o^C overnight. After several rinses in PBS, cells were incubated with fluorescence-conjugated donkey anti-mouse, anti-sheep, or anti-rabbit IgG (1:1000, Invitrogen) for 1 h at 22–24^o^C. Primary antibodies included mouse anti-NSF (1:200, Abcam) and anti-TAU (1:200, Abcam). Imaging was similar to SypH imaging. mCherry fluorescence imaging was performed simultaneously to identify cells transfected with Cre/mCherry.

#### SynaptopHluorin imaging in hippocampal neurons

Action potentials were evoked by multiple 1-ms pulses (20 mA) delivered at different frequencies through a platinum electrode. The bath solution contained (in mM): 119 NaCl, 2.5 KCl, 2 CaCl_2_, 2 MgCl_2_, 25 HEPES (buffered to pH 7.4), 30 glucose, 0.01 6-cyano-7-nitroquinoxaline-2, 3-dione (CNQX), and 0.05 D, L-2-amino-5-phosphonovaleric acid. We heated the culture chamber using a temperature controller (TC344B, Warner Instruments, Hamden, CT). Imaging was performed after the culture was at 34–37°C for 15–30 min. The temperature was verified with another small thermometer (BAT-7001H, Physitemp Instruments, Clifton, NJ) in the chamber. SypH images were acquired at 10 Hz using Nikon A1 confocal microscope (Objective: 60×, 1.4 NA), and analyzed with Nikon software. All boutons showing fluorescence increases were analyzed (region of interest: 2 × 2 μm). Each data group was obtained from at least three batches of cultures.

#### Electron microscope images, data collection and analysis of hippocampal neurons

Hippocampal cultures were fixed with 4% glutaraldehyde (freshly prepared, Electron microscopy sciences, Hatfield, PA) in 0.1 M Na-cacodylate buffer solution containing for at least 1 h at 22–24°C and stored in 4°C refrigerator overnight. The next day, cultures were washed with 0.1 M cacodylate buffer and treated with 1% OsO_4_ in cacodylate buffer for 1 h on ice, and 0.25% uranyl acetate in acetate buffer at pH 5.0 overnight at 4°C, dehydrated with ethanol, and embedded in epoxy resin. Thin sections were counterstained with uranyl acetate and lead citrate then examined in a JEOL200CX TEM. Images were collected with a CCD digital camera system (XR-100; AMT) at a primary magnification of 10,000–20,000×. Synapses were selected based on the structural specialization including synaptic vesicle clustering, synaptic cleft and the postsynaptic density.

#### Chromaffin cell culture and transfection

The primary bovine adrenal chromaffin cell culture has been described previously.^23^^,^^26^^,^^49^ We purchased fresh adrenal glands (from 21–27 months old bovines of either sex) from a local slaughterhouse (J. W. Treuth & Sons Inc., 328 Oella Ave, Catonsville, MD 21228; web site: https://www.jwtreuth.com). The glands were immersed in pre-chilled Locke’s buffer on ice for transportation to the lab. The Locke’s buffer contained (mM): NaCl, 145; KCl, 5.4; Na_2_HPO_4_, 2.2; NaH_2_PO_4_, 0.9; glucose, 5.6; HEPES, 10 (pH 7.3, adjusted with NaOH). The glands were perfused with Locke’s buffer, then infused with Locke’s buffer containing collagenase P (1.5 mg/ml, Roche), trypsin inhibitor (0.325 mg/ml, Sigma) and bovine serum albumin (5 mg/ml, Sigma), and incubated at 37**°**C for 20 min. The digested medulla was minced in Locke’s buffer, and filtered through a 100 nm nylon mesh. The filtrate was centrifuged (48 × g, 5 min), re-suspended in Locke’s buffer and re-centrifuged until the supernatant was clear. The final cell pellet was re-suspended in pre-warmed DMEM medium (Gibco) supplemented with 10% fetal bovine serum (Gibco).

Cells were transfected by electroporation using Basic Primary Neurons Nucleofector Kit (Lonza), according to the manufacturer’s protocol and plated onto poly-L-lysine (0.005 % w/v, Sigma) and laminin (4 μg/ml, Sigma) coated glass coverslips (Neuvitro). The cells were incubated at 37°C with 9% CO_2_ and used within 48 h.^49^

#### Fluorescent dyes and plasmids for chromaffin cells

For FFN511 (Abcam) imaging, cells were bathed with FFN511 (5–10 μM) in 37°C incubator for 20 min and images were performed after washing out FFN511 in the bath solution. Atto 655 (A655, Sigma) was included in the bath solution at the concentration of 30 μM. PH-EGFP (phospholipase C delta PH domain attached with EGFP) was obtained from Dr. Tamas Balla. PH-mNeonGreen (PH_G_) was created by replacing the EGFP tag of PH-EGFP with mNeonGreen (Allele Biotechnology).^49^

For knockdown of endogenous NSF in bovine chromaffin cells, a siRNA duplex for bovine NSF (5′- CCAGAUUGUCGAUGUGUUU-3′) labeled with cyanine 3 (si-NSF) and scrambled control siRNA (si-Ctrl) labeled with cyanine 3 were purchased from Sigma-Aldrich. For rescue experiments, si-NSF and a plasmid containing wild-type NSF and mCherry (for recognition of the transfected cell) (Addgene #84334) were transfected into chromaffin cells. The cDNA encoding NSF_E329Q_ (Genecopoeia) was subcloned into EBFP2-C1 (Addgene #54665), where EBFP2 was used to recognize transfected cells.

#### Western blot

Total protein was extracted from cultured chromaffin cells or hippocampal cultures using RIPA buffer containing protease inhibitor cocktail (Millipore Sigma). Equal amounts of proteins, determined by BCA protein assay (Invitrogen) were loaded onto 4%–12% Bis-Tris gel (Invitrogen). Proteins were transferred onto PVDF membrane and immunoblotted with the indicated primary antibodies at 4°C overnight. Membranes were incubated with HRP-labeled secondary antibodies at 22–24^o^C for 2 h and visualized using Bio-Rad ChemiDoc Imaging System. Primary antibodies included anti-NSF (1:2000, Abcam), mouse anti-CHC (1:500, Abcam), rabbit anti-dynamin (1:1000, Cell Signaling Technology), mouse anti-AP2 (1:1000, ThermoFisher Scientific), and β-actin (1:3000; Abcam).

#### Electrophysiological recording at chromaffin cells

The method has been described before.^23^^,^^26^^,^^49^ At room temperature (20–22°C), whole-cell voltage-clamp and capacitance recordings were performed with an EPC-10 amplifier together with the software lock-in amplifier (PULSE 8.74, HEKA, Lambrecht, Germany). The holding potential was –80 mV. For capacitance measurements, the frequency of the sinusoidal stimulus was 1000 Hz with a peak-to-peak voltage ≤ 50 mV. The bath solution contained (mM): 125 NaCl, 10 glucose, 10 HEPES, 5 CaCl_2_, 1 MgCl_2_, 4.5 KCl, 0.001 TTX and 20 TEA, pH 7.3 adjusted with NaOH. The pipette (2–4 MΩ) solution contained (mM) 130 Cs-glutamate, 0.5 Cs-EGTA, 12 NaCl, 30 HEPES, 1 MgCl_2_, 2 ATP, and 0.5 GTP, pH 7.2 adjusted with CsOH. These solutions pharmacologically isolated calcium currents. For stimulation, we used a 1-s depolarization from the holding potential of –80 mV to +10 mV (depol_1s_). We used this stimulus because it induces robust exo-endocytosis as reflected in capacitance recordings. Since prolonged whole-cell recording slows down endocytosis, we limited to 1 depol_1s_ per cell.

#### Confocal imaging at chromaffin cells

Imaging of PH_G_, FFN511, and A655 was performed with an inverted confocal microscope (TCS SP5II, Leica, Germany, 100× oil objective, numerical aperture: 1.4).^25^^,^^49^ PH_G_ was excited by a tunable white light laser at 515 nm (laser power set at ∼1–4 mW); FFN511 was excited by an Argon laser at 458 nm (laser power set at ∼2–4 mW); A655 was excited by an HeNe laser at 633 nm (laser power set at ∼12–15 mW); their fluorescence was collected at 520–600 nm, 465–510 nm, and 650–800 nm, respectively. Confocal imaging area was ∼70–160 μm^2^ at the XY plane with a fixed Z-axis focal plane ∼100–200 nm above the cell-bottom membrane (XY/Z_fix_ scanning). Images were collected every 40–80 ms at 40–60 nm per pixel.

#### Fusion modes, close-fusion and pre-close detection with confocal microscopy

Full-fusion was identified as the sudden appearance of PH_G_ spot or ring together with the sudden appearance of an A655 spot, due to PH_G_ and A655 diffusion from the plasma membrane (PM) and the bath into the fusion-generated Ω-profile (fs-Ω, Figure 6) at cell-bottom. FFN511 (pre-loaded in vesicles) fluorescence (F_FFN_) decrease concurrently at the same spot as PH_G_ fluorescence (F_PH_) and A655 fluorescence (F_655_) increased, while measurements was made for estimation of FFN511 release rate. The fusion pore closes at ∼0.05–30 s later (close-fusion, Figure 6), maintains an open pore (stay-fusion, Figure 6), or shrinks to merge with the plasma membrane (shrink-fusion, Figure 6).^24^^,^^25^^,^^31^^,^^49^

Close-fusion was detected as F_655_ (strongly excited) dimming due to pore closure that prevented bath fluorescent A655 from exchanging with bleached A655 in vesicle, while F_PH_ (weakly excited) sustained or decayed with a delay that reflected vesicle pinch off (Figure 6); stay-fusion was detected as sustained F_655_ and F_PH_ (Figure 6); shrink-fusion was detected as parallel increases and decreases of F_655_ and F_PH_ (Figure 6).

Pre-close was detected with spot F_655_ bleaching with constant F_PH_, due to fusion pore closure of pre-Ω by strong excitation (Figure 5). It is not due to a narrow pore smaller than A655 molecule size, because after spot dimming, bath application of an acid solution cannot quench the pH-sensitive VAMP2-EGFP or VAMP2-pHluorin overexpressed at the same spot, indicating that the spot is impermeable to H^+^ or OH^-^, the smallest molecules, and thus is closed.^23^

### Quantification and statistical analysis

#### Data collection and quantifications

For membrane capacitance (Cm) measurements at calyces, each group of data were from 7–12 calyces, with one calyx recorded per mouse (7–12 mice of either sex). The exact numbers of calyces and mice for each experimental group are indicated in the corresponding figure legends. Cm was measured within 10 min after break-in to avoid rundown. The first 0.25 s Cm trace after stimulation was not used (and thus not shown in Figures 1 and 2) to avoid capacitance artifact contamination.^9^^,^^13^^,^^46^^,^^47^

In each pHluorin imaging experiment, 20–30 synaptic boutons exhibiting stimulus-evoked increases in SypH fluorescence (F_SypH_) were analyzed. Square regions of interest (ROIs; 2 μm × 2 μm) were manually defined over individual boutons. Approximately one to three experiments were performed per culture, with each culture prepared from 3–6 mice. Data for each experimental group were obtained from at least four independent batches of cultures (4–12 cultures). F_SypH_ was normalized to the baseline F_SypH_ before stimulation (baseline F_SypH_ was normalized as 100%). Rate_decay_ (the initial rate of F_SypH_ decay) was measured from F_SypH_ in the first 4 – 10 s after stimulation.

For electron microscopy, hippocampal synapses were identified based on characteristic ultrastructural specializations, including synaptic vesicle clustering, the presence of a synaptic cleft, and postsynaptic density. Each dataset was derived from 120–122 synaptic profiles collected from 18 mice across three independent cultures.

For chromaffin cell experiments, each dataset was obtained from at least four independent primary chromaffin cell cultures. Each culture was prepared using at least three adrenal glands collected from two individual bovines. Cm and ICa were collected within the first 2 min after the break-in to avoid rundown of endocytosis and Ca^2+^ influx, which appear as a gradual decline in both,^26^^,^^50^ and were analyzed using Igor (WaveMetrics). Confocal images were analyzed with LAS X (Leica) and ImageJ. Fluorescence intensity was measured at each frame within the fluorescence spot. Cells with fewer than five fusion events were excluded to avoid biasing the dataset.

#### Statistical tests

Data are presented as mean ± s.e.m. The number of replicates (n) is specified in the Results and figure legends, where n represents the number of cells, fusion events, or independent experiments as indicated. Statistical analyses were performed using unpaired two-tailed Student’s *t*-tests. Statistical significance is denoted as follows: *p* < 0.05 (∗), *p* < 0.01 (∗∗), and *p* < 0.001 (∗∗∗).
