# Supplementary material for: NSF is required for diverse endocytic modes by promoting fusion and fission pore closure in secretory cells
Source: iScience. 2026 Mar 27;29(5):115510. doi: 10.1016/j.isci.2026.115510 (PMC13091539; doi:10.1016/j.isci.2026.115510)
Supplement: Document S1. Figures S1–S7 [file mmc1.pdf]

iScience, Volume *29*

Supplemental information

NSF is required for diverse endocytic modes by promoting fusion and fission pore closure in secretory cells

Xin-Sheng Wu, Tao Sun, Bo Shi, Sunghoon Lee, Zheng Zhang, Lisi Wei, Xin Wang, Maryam Molakarimi, Sue Han, Aaron Uy, Lin Gan, and Ling-Gang Wu

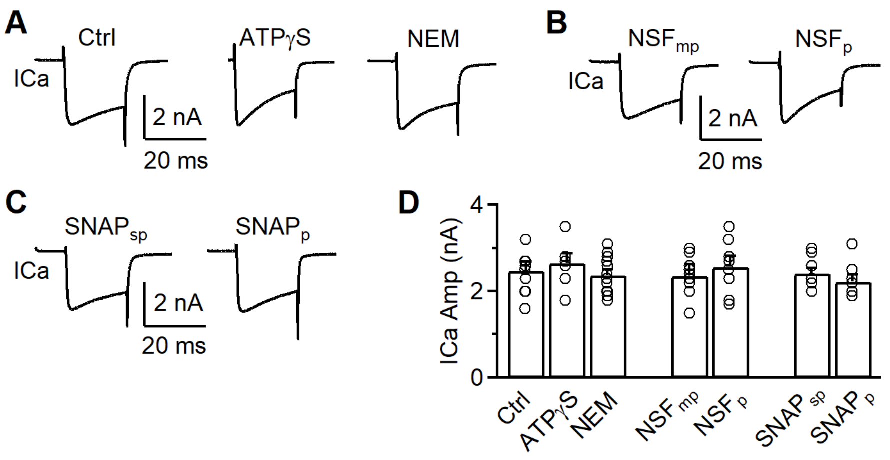

Figure S1. NSF blockers do not affect calcium currents evoked by 20 ms stimuli; related to Figure 1.

(A) Sampled traces (single trace) of similar calcium currents (ICa) induced by a 20 ms depolarization from –80 to +10 mV (depol$_{20ms}$) with a pipette solution containing a control solution (Ctrl), 4 mM ATPγS (replacing ATP) or 1 mM NEM. Data were taken in 4–10 min after whole-cell break-in. Vertical scale bar: 2 nA; horizontal scale bar: 20 ms. Scale bars apply to all traces in A.

(B) Similar to panel A, but with NSF$_{mp}$ (1 mM) or NSF$_{p}$ (1 mM).

(C) Similar to panel A, but with SNAP$_{sp}$ (1 mM) or SNAP$_{p}$ (1 mM).

(D) Similar ICa amplitudes (ICa Amp) induced by depol$_{20ms}$ at 4–10 min after break-in with a pipette containing the control solution (Ctrl, n = 11 calyces, from 5 male and 6 female rats), ATPγS (4 mM, n = 6 calyces, from 3 male and 3 female rats), NEM (1 mM, n = 12 calyces, from 6 male and 6 female rats), NSF$_{mp}$ (1 mM, n = 10 calyces, from 5 male and 5 female rats), NSF$_{p}$ (1 mM, n = 9 calyces, from 4 male and 5 female rats), SNAP$_{sp}$ (1 mM, n = 7 calyces, from 3 male and 4 female rats) or SNAP$_{p}$ (1 mM, n = 8 calyces, from 4 male and 4 female rats). Data are presented as mean + s.e.m., with each circle representing the data from a single calyx.

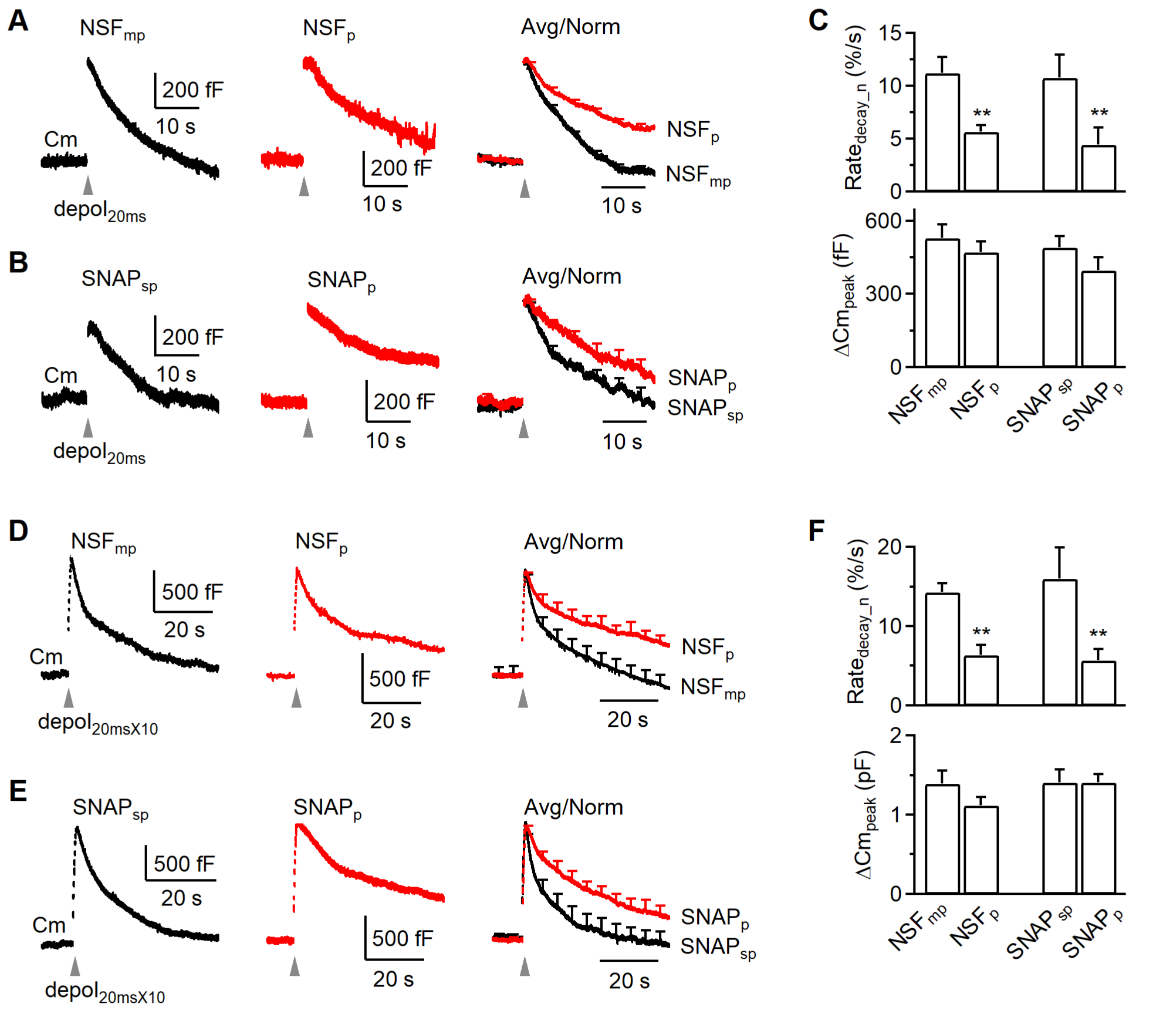

Figure S2. NSF is involved in slow and rapid endocytosis at calyces: results observed 2–4 min after whole-cell break-in; related to Figures 1 and 2.

(A) Sampled membrane capacitance changes (Cm) induced by $depol_{20ms}$ (gray arrowheads) at 2–4 min after break-in with a pipette containing NSF_{mp} (1 mM, black, n = 10 calyces, from 5 male and 5 female rats) or NSF_p (1 mM, red, n = 9 calyces, from 4 male and 5 female rats). Traces on the left and middle are single traces, whereas traces on the right are averaged traces (Avg/Norm) with the amplitude normalized for comparison of the decay. Traces on the right were expressed as mean + s.e.m. every 5 s (applies also to panels B, D, and E). Vertical scale bars: 200 fF; horizontal scale bars: 10 s.

(B) Similar to panel A, but with $SNAP_{sp}$ (1 mM, black, n = 7 calyces, from 3 male and 4 female rats) or $SNAP_p$ (1 mM, red, n = 8 calyces, from 4 male and 4 female rats). Vertical scale bars: 200 fF; horizontal scale bars: 10 s.

(C) The $Rate_{decay\_n}$ and ΔCm_{peak} induced by $depol_{20ms}$ at 2–4 min after break-in with a pipette containing NSF_{mp} (1 mM, n = 10 calyces, from 5 male and 5 female rats), NSF_p (1 mM, n = 9 calyces, from 4 male and 5 female rats), $SNAP_{sp}$ (1 mM, n = 7 calyces, from 3 male and 4 female rats) or $SNAP_p$ (1 mM, n = 8 calyces, from 4 male and 4 female rats). Data are presented as mean + s.e.m. **: $p < 0.01$(*t*-test).

(D-F) Similar arrangement as panels A–C (including the calyx number), respectively, except that the stimulus was $depol_{20msX10}$. Vertical scale bars: 500 fF; horizontal scale bars: 20 s. **: $p < 0.01$ (*t*-test).

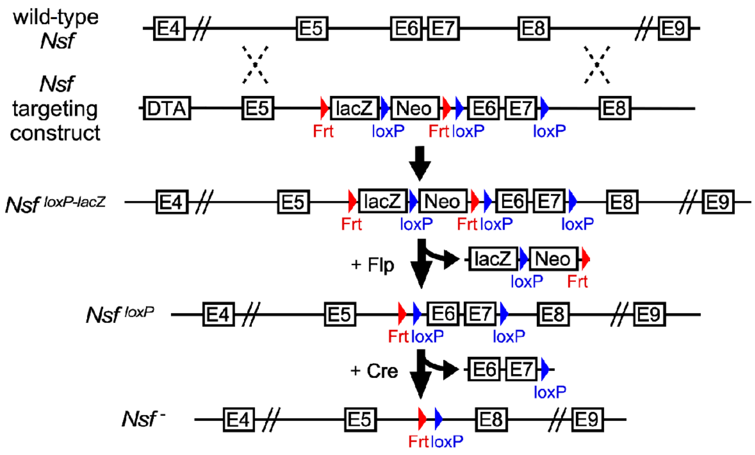

Figure S3. Generation of the *NSF* conditional knockout allele; related to Figures 3 and 4.
The *NSF* targeting construct was generated by the Knockout Mouse Project (KOMP). A 5.8 kb NSF genomic fragment containing exon 5 and a 3.8 kb fragment containing exon 8 were used as the 5′ and 3′ homologous arms, respectively. A synthetic 7.1 kb fragment containing an Frt-flanked *lacZ* reporter gene and neomycin (Neo) cassette, as well as loxP-flanked exons 6 and 7, were inserted between the 5' and 3' homologous arms. Targeted *NSF*$^{loxP\text{-}lacZ}$ embryonic stem cells (ESCs) on C57BL/6N background, generated by and purchased from the KOMP repository, were injected into C57BL/6J blastocysts to generate chimeric founder mice. The founder mice were bred with wild-type C57BL/6J to generate *NSF*$^{loxP\text{-}lacZ}$ heterozygotes. These mice were subsequently crossed with Rosa26-Flpe mice (Jackson Laboratory, stock# 009086) to remove the *lacZ* and Neo cassette, generating *NSF*^{loxP} mice. Crossing *NSF*^{loxP} mice with Cre-expressing mice resulted in *NSF*$^{-/-}$ mice by deleting loxP-flanked exons 6 and 7, causing a frameshift and premature termination codon in exon 8.

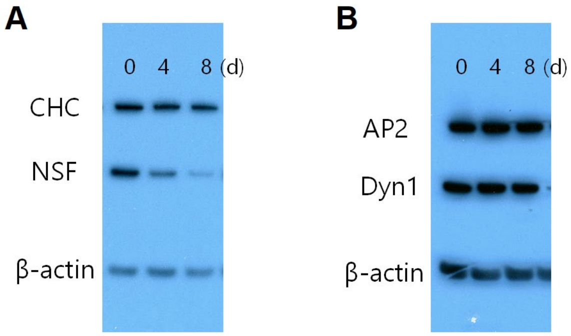

Figure S4. Western blot of NSF knockdown in hippocampal neurons; related to Figure 3A and B.

(A) A representative Western blot shows endogenous clathrin heavy chain (CHC), NSF, and β-actin levels in hippocampal neurons at Days 0, 4, and 8 after Cre-4-OHT treatment. β-actin served as the loading control.

(B) Similar to panel A, but showing AP2, dynamin 1 (Dyn1), and β-actin levels.

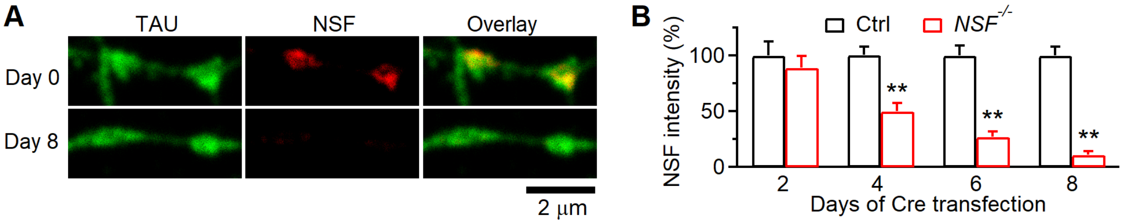

Figure S5. Immunostaining showing deletion of NSF in the *NSF*$^{-/-}$ hippocampal culture; related to Figures 3 and 4.

(A) Immunostaining of TAU (labelling the axon) and NSF in day 0 and day 8 after Cre-mCherry transfection to *NSF*$^{LoxP/LoxP}$ hippocampal cultures. Horizontal scale bar: 2 μm.

(B) NSF immunostaining intensity in day 2, day 4, day 6, and day 8 after Cre-mCherry transfection to *NSF*$^{LoxP/LoxP}$ hippocampal cultures (red, *NSF*$^{-/-}$, 3 cultures) or wild-type hippocampal cultures (black, Ctrl, 3 cultures). Data are presented as mean + s.e.m. **: $p < 0.01$(*t*-test).

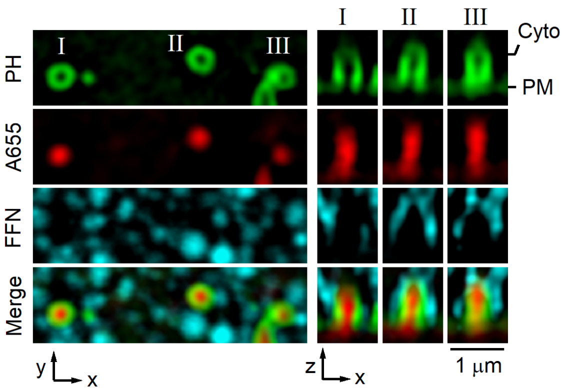

Figure S6. Confocal images of PH (or PH_G), A655, and FFN511 (near cell-bottom) showing pre-spot I, II and III at the XY- (left, ring-shape) and XZ-plane (right, W-shape). Horizontal scale bar: 1 μm. Data taken from Ref. [S1] with permission. It is related to Figures 5 and 6.

A

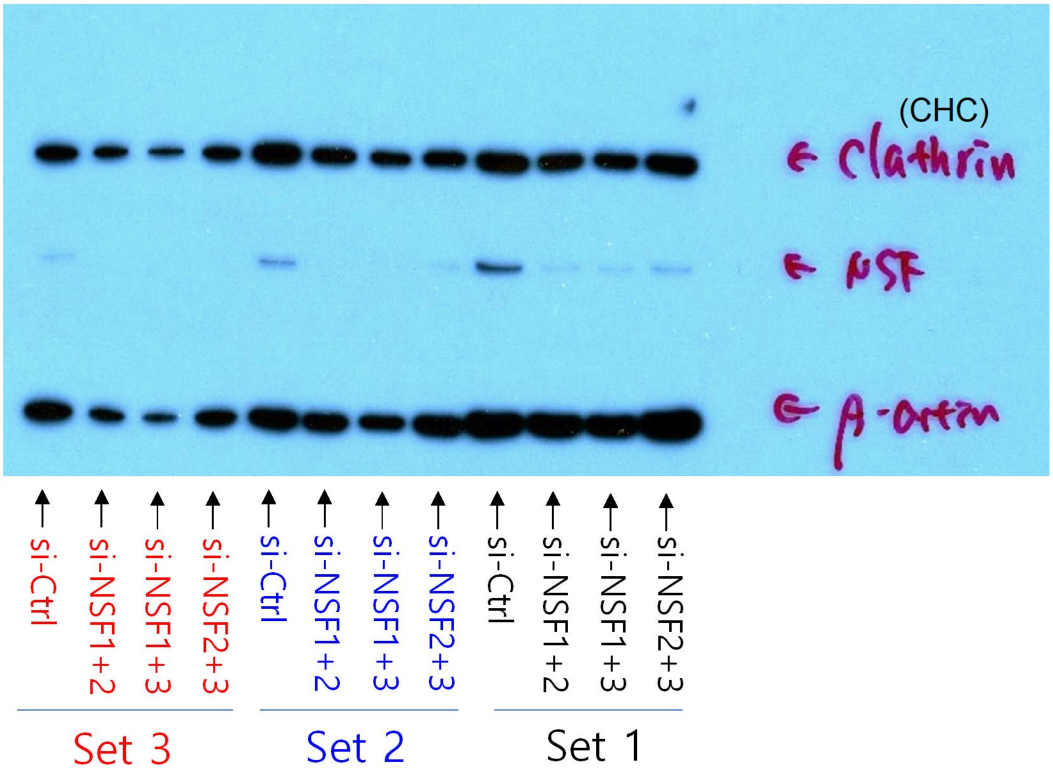

B

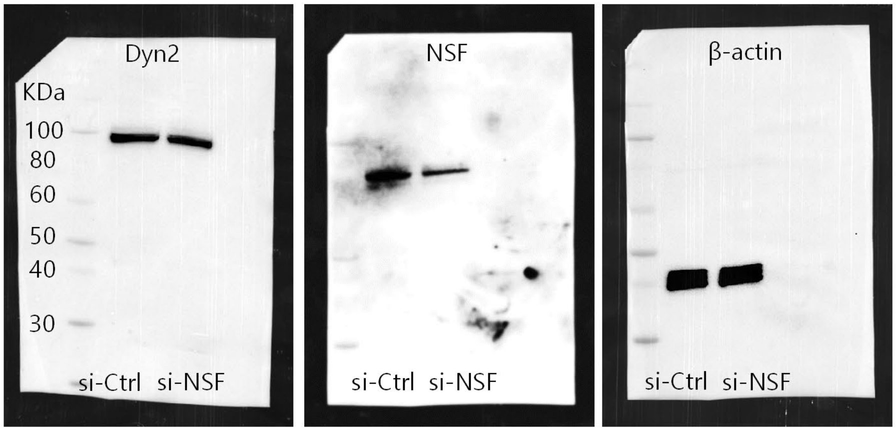

Figure S7. Western blot of chromaffin cells transfected with si-NSF, related to Figures 5 and 6.

(A) Three representative Western blot sets (Sets 1–3) show endogenous NSF, clathrin heavy chain (CHC), and β-actin levels in chromaffin cells at Day 2 after transfection with si-NSF1 + 2, si-NSF1 + 3, or si-NSF2 + 3. β-actin served as the loading control.

(B) Representative Western blot shows endogenous dynamin 2 (Dyn2), NSF, and β-actin levels in chromaffin cells at Day 2 after transfection with si-NSF1 + 2 + 3.

Supplemental references

1. Wei, L., Guo, X., Haimov, E., Obashi, K., Lee, S.H., Shin, W., Sun, M., Chan, C.Y., Sheng, J., Zhang, Z., et al. (2024). Clathrin mediates membrane fission and budding by constricting membrane pores. Cell Discov *10*, 62. 10.1038/s41421-024-00677-w.
